# Evaluating the Causal Effects of TIMP-3 on Ischaemic Stroke and Intracerebral Haemorrhage: A Mendelian Randomization Study

**DOI:** 10.3389/fgene.2022.838809

**Published:** 2022-04-04

**Authors:** Linxiao Xiao, Xuelun Zou, Yan Liang, Yuxiang Wang, Lang Zeng, Jianhuang Wu

**Affiliations:** ^1^ Department of Spine Surgery and Orthopaedics, Xiangya Hospital, Central South University, Changsha, China; ^2^ Department of Neurology, Xiangya Hospital, Central South University, Changsha, China; ^3^ National Clinical Research Center for Geriatric Disorders, Xiangya Hospital, Central South University, Changsha, China

**Keywords:** ischaemic stroke, intracerebral haemorrhage, genome-wide association study, mendelian randomisation, TIMP-3

## Abstract

**Aim:** Since tissue inhibitors of matrix metalloproteinase 3 (TIMP-3) was reported to be a potential risk factor of atherosclerosis, aneurysm, hypertension, and post-ischaemic neuronal injury, it may also be a candidate risk factor of stress. Therefore, this study was designed to explore the causal role of TIMP-3 in the risk of ischaemic stroke (IS) and intracerebral haemorrhage (ICH), which are the two main causes of stress via this Mendelian Randomisation (MR) study.

**Methods:** The summarised data of TIMP-3 level in circulation was acquired from the Cooperative Health Research in the Region of Augsburg public database and the outcome of IS and ICH was obtained from genome-wide association studies conducted by MEGASTROKE and the International Stroke Genetics Consortium, respectively. Five statistical methods including inverse-variance weighting, weighted-median analysis, MR-Egger regression, MR Pleiotropy RESidual Sum and Outlier test, and MR-Robust Adjusted Profile Score were applied to evaluate the causal role of TIMP-3 in the occurrence of IS and ICH. Inverse-variance weighting was applied for assessing causality. Furthermore, heterogeneity and pleiotropic tests were utilised to confirm the reliability of this study.

**Results:** We found that TIMP-3 could be a positively causal relationship with the incidence of IS (OR = 1.026, 95% CI: 1.007–1.046, *p* = 0.0067), especially for the occurrence of small vessel stroke (SVS; OR = 1.045, 95% CI: 1.016–1.076, *p* = 0.0024). However, the causal effects of TIMP-3 on another IS subtype cardioembolic stroke (CES; OR = 1.049, 95% CI: 1.006–1.094, *p* = 0.024), large artery stroke (LAS; OR = 1.0027, 95% CI: 0.9755–1.0306, *p* = 0.849) and ICH (OR = 0.9900, 95% CI: 0.9403–1.0423, *p* = 0.701), as well as ICH subtypes were not observed after Bonferroni corrections (*p* = 0.00714).

**Conclusion:** Our results revealed that high levels of circulating TIMP-3 causally increased the risk of developing IS and SVS, but not CES, LAS, ICH, and all ICH subtypes. Further investigation is required to elucidate the underlying mechanism.

## Introduction

Although the incidence of stroke has declined in high-income countries in the 21st century, the number of patients with stroke has not decreased to a discernible degree (Li et al., 2020). More importantly, the prevalence of ischaemic stroke (IS), the most common type of stroke, is estimated to rise by 27% over the next 40 years (Wafa et al., 2020). In addition to IS, intracerebral haemorrhage (ICH), another life-threatening type of stroke, has attracted attention due to its high mortality and disability rates (Lioutas et al., 2020; GBD, 2021). The 30-days case fatality rate of ICH is as high as 40% and the annual fatality rate is 54%. Furthermore, only 12–39% of survivors achieve long-term functional independence (An et al., 2017). The high prevalence of stroke increases the disease burden on the population (GBD, 2019; GBD, 2021). Altogether, early prevention and treatment of these two types of strokes is required to improve clinical outcomes and decrease the healthcare burden.

The first pathological changes of IS and ICH appear as cerebrovascular disease evidenced by damaged blood vessels and the destruction of the blood-brain barrier due to degradation of the extracellular matrix (ECM) and disintegration of intercellular connections by matrix metalloproteinases (MMPs) (Castro and Tanus-Santos, 2013; Kurzepa et al., 2014; Ungvari et al., 2017; Li et al., 2019a; Li et al., 2019a). The release of MMPs and their pathogenic effects on stroke are regulated by tissue inhibitors of matrix metalloproteinases (TIMPs) (Pushpakumar et al., 2013; Li et al., 2019a). Among TIMPs, TIMP-3 is closely associated with atherosclerosis (Di Gregoli et al., 2017), aneurysm (Di Gregoli et al., 2017), hypertension (Higuchi et al., 2007), and post-ischaemic neuronal injury (Wallace et al., 2002), but its relationship with IS and ICH has not been confirmed.

Mendelian randomisation (MR) is a novel epidemiological analysis method utilising genetic variation to determine the causal associations between risk factors and outcomes (Emdin et al., 2017). Compared to traditional observational studies, MR has the advantages of controlling the influence confounding factors have on the assessment of causality and reducing the influence of reverse causality (Zheng et al., 2017; Lee and Lim, 2019). This investigation aimed to reveal the causal role of TIMP-3 in the risk of developing IS and ICH by performing a two-sample MR study. In addition, we also analysed the causal relationship between TIMP-3 and the main subtypes of IS (large artery stroke [LAS], small vessel stroke [SVS], cardioembolic stroke [CES]), and ICH (lobar intracerebral haemorrhage [LICH] and non-lobar intracerebral haemorrhage [NLICH]).

## Materials and Methods

### Data Sources and Genetic Instruments

In this MR study, the summary-level data of TIMP-3 was obtained from a cohort study including approximately 1,000 participants available in the KORA (Cooperative Health Research in the Region of Augsburg) public database (Suhre et al., 2017). All participants of this GWAS were of European descent. The IS data were extracted from a genome-wide association meta-analysis of 29 studies, comprising approximately 440,328 participants (34,217 cases and 406,111 non-cases) of European descent (Malik et al., 2018). This meta-analysis identified 32 loci related to the risk of stroke and detected a total of 7,537,579 single nucleotide polymorphisms (SNPs). The subtypes of IS were derived from this meta-analysis. The SVS cohort had 198,048 individuals and 6,150,261 related SNPs. The CES cohort had 211,763 individuals and 7,954,834 related SNPs. The LAS cohort had 150,765 individuals and 7,992,739 related SNPs. The statistical information of ICH was acquired from a genome-wide association meta-analysis performed by the International Stroke Genetics Consortium and included 3,026 Europeans (Woo et al., 2014). The ICH cohort was further divided into LICH and NLICH cohorts.

SNPs were selected as instrumental variables from the GWAS database of *TIMP-3* variants and used to investigate the causal relationship. Based on published research (Sun et al., 2020), we defined the inclusion criteria of SNPs as those with *p* values less than 1 × 10^–5^. In addition, we clumped SNPs by setting *r* ^2^ **>** 0.1 to exclude the influence of linkage disequilibrium on the results (Sun et al., 2020).

### Mendelian Randomisation Analysis

We conducted two-sample MR analyses to explore the potential causal role of serum TIMP-3 in stroke outcomes (IS, ICH, and their subtypes). The traditional evaluation method of inverse-variance weighting (IVW) was used as the primary method to evaluate causality in this study (Jia et al., 2021). Accurate causality can be obtained in the presence of directional pleiotropy using the IVW approach (Burgess et al., 2015). Depending on the heterogeneity, we decided whether the random-effect IVW method or fixed-effect IVW method should be used (Jia et al., 2021). The heterogeneity of instrumental variables was assessed using Cochran’s Q statistic (Greco et al., 2015). Weighted median estimation (WME) can provide a consistent assessment if more than 50% of the weights for the SNPs come from valid SNPs (Bowden et al., 2016a). The MR-Egger regression method is another method that can assist in estimating the causal relationship, although some included SNPs were atypical (Bowden et al., 2015; Bowden et al., 2016b); however, its statistical power is relatively weak (Bowden et al., 2015). In addition to assessing the causal relationship, MR-Pleiotropy RESidual Sum and Outlier (MR-PRESSO) has the advantage of detecting outliers of instrumental variables and providing evidence of directional pleiotropy (Verbanck et al., 2018). MR-Robust Adjusted Profile Score (MR. RAPS) can overcome the bias of weak instrumental variables and the effects of systematic and idiosyncratic pleiotropy to obtain a robust causal assessment (Zhao et al., 2018).

In the sensitivity analysis, the Egger regression method provided a way to detect pleiotropy, which is expressed by the value of the MR-Egger intercept. If the intercept is equal to 0 (*p* ≥ 0.05), it suggests that there is no pleiotropy (Hemani et al., 2018). Furthermore, the MR-PRESSO global test can also examine directional pleiotropy (Verbanck et al., 2018). If pleiotropy exists in instrumental variables, it implies that some other potential pathways may affect the pathway from instrumental variables and exposure factors to the outcome, which does not conform to the third core hypothesis of MR analysis [see Section 2.3]. The MR-PRESSO outlier test is a detection method similar to leave-one-out. It can eliminate all included SNPs one by one, correct the bias caused by outliers, and show robust causal estimation (Emdin et al., 2017; Ong and MacGregor, 2019).

### Three Core Assumptions

**FIGURE 1:**
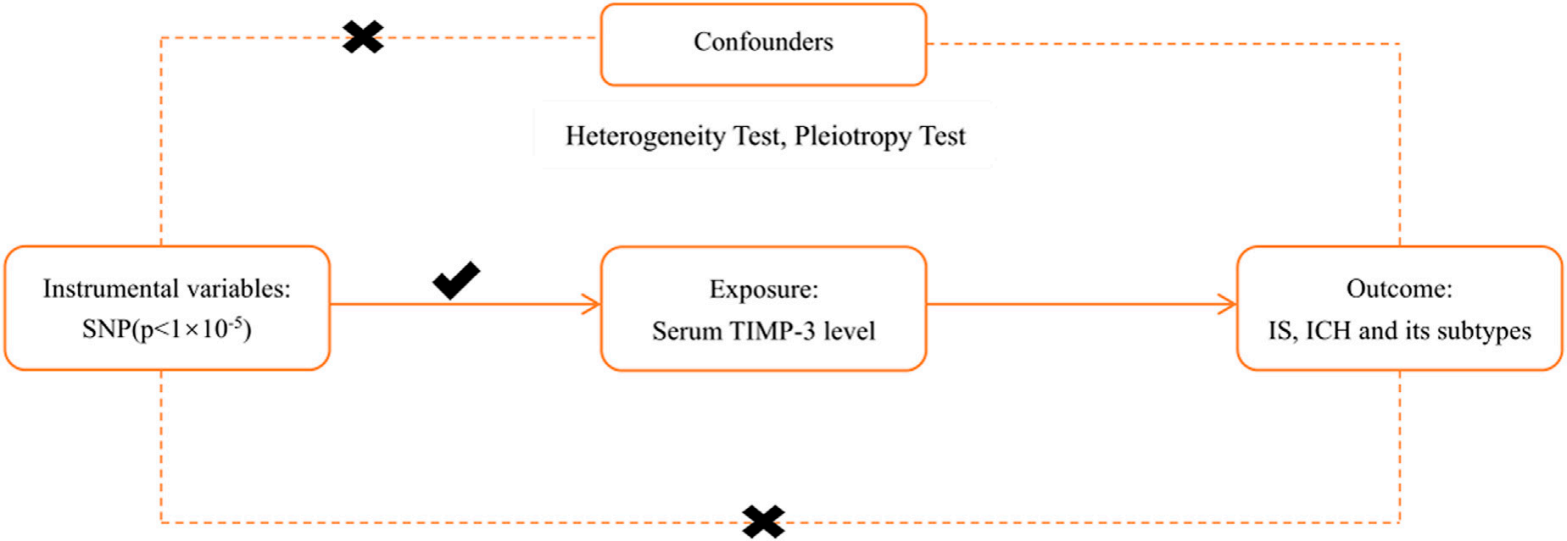
The principal diagram for the Mendelian randomization study. IVW = inverse-variance-weighted, SNP = single nucleotide polymorphism, IS = ischemic stroke, LAS = large vessel ischemic stroke, CES = cardioembolic ischemic stroke, SVS = small vessel ischemic stroke, ICH = intracerebral hemorrhage. TIMP-3 = Tissue inhibitors of matrix metalloproteinases 3.

To make rational use of MR analysis and improve the efficiency and accuracy of the study, all Mendelian randomisation studies must meet three core assumptions before they are conducted (Figure 1) (Emdin et al., 2017). First, instrumental variables (SNPs) should be closely related to exposure factors (TIMP-3). As shown in Supplementary Table S1, all included SNPs were statistically significantly related to TIMP-3. Second, instrumental variables (SNPs) should not be associated with any confounder which correlates with exposure (TIMP-3) or outcomes (IS, ICH). We defined confounders as carotid plaque formation, neuroinflammation, atrial fibrillation, coronary artery disease, blood pressure, type II diabetes, hypertension, systolic and diastolic blood pressure, high density lipoprotein levels, low density lipoprotein levels, and triglyceride levels (Chauhan and Debette, 2016; Cai et al., 2019). We used the PhenoScanner website (Staley et al., 2016) to query all related phenotypes of the included SNPs and found no phenotypes related to the confounding factors (*p* < 1 × 10^–5^) (Cai et al., 2019). Finally, instrumental variables should not be other methods related to an outcome other than the pathway of exposure (Supplementary Table S2). We conducted strict tests for heterogeneity and pluripotency and found no other pathways that were statistically significant; that is, there were no other pathways from SNPs to the outcome (IS and ICH) other than the exposure (TIMP-3) (Emdin et al., 2017).

### Statistical Analysis

The R studio software with two packages, “Mendelian Randomisation” and “TwoSampleMR”, was used to conduct the MR analysis. The principal analyses for TIMP-3, IS, and ICH were performed using the random-effects model IVW approach. The statistical tests to estimate causal relationships were regarded as statistically significant at a Bonferroni corrected *p* < 0.007 (0.05/7; *p* = 0.05 adjusted for seven tests). *p*-value was set at 0.05 for statistical significance for both pleiotropy and heterogeneity tests.

## Results

### Selection of Instrumental Variables

As shown in Supplementary Table S1, from the GWAS data of *TIMP-3*, we identified 10 statistically significant instrumental variables, namely rs135150, rs137487, rs17303757, rs2097326, rs241890, rs34843069, rs3788495, rs5754249, rs5754266, and rs9422881. However, we could not find rs17303757 in the IS GWAS data. rs17303757 or rs34843069 in SVS data or rs17303757 or rs5754266 in ICH subtypes data. Therefore, IS had only nine valid instrumental variables while SVS, ICH, and the ICH subtypes had only eight instrumental variables. Although a more relaxed *p*-value threshold (*p* < 1 × 10^–5^) for the inclusion of instrumental variables was adapted, as the F statistic was >10 (Supplementary Table S1), the potential of weak instrumental variable bias was ruled out.

### The Causal Role of TIMP-3 Was Significantly Positively Associated With Ischaemic Stroke

**FIGURE 2:**
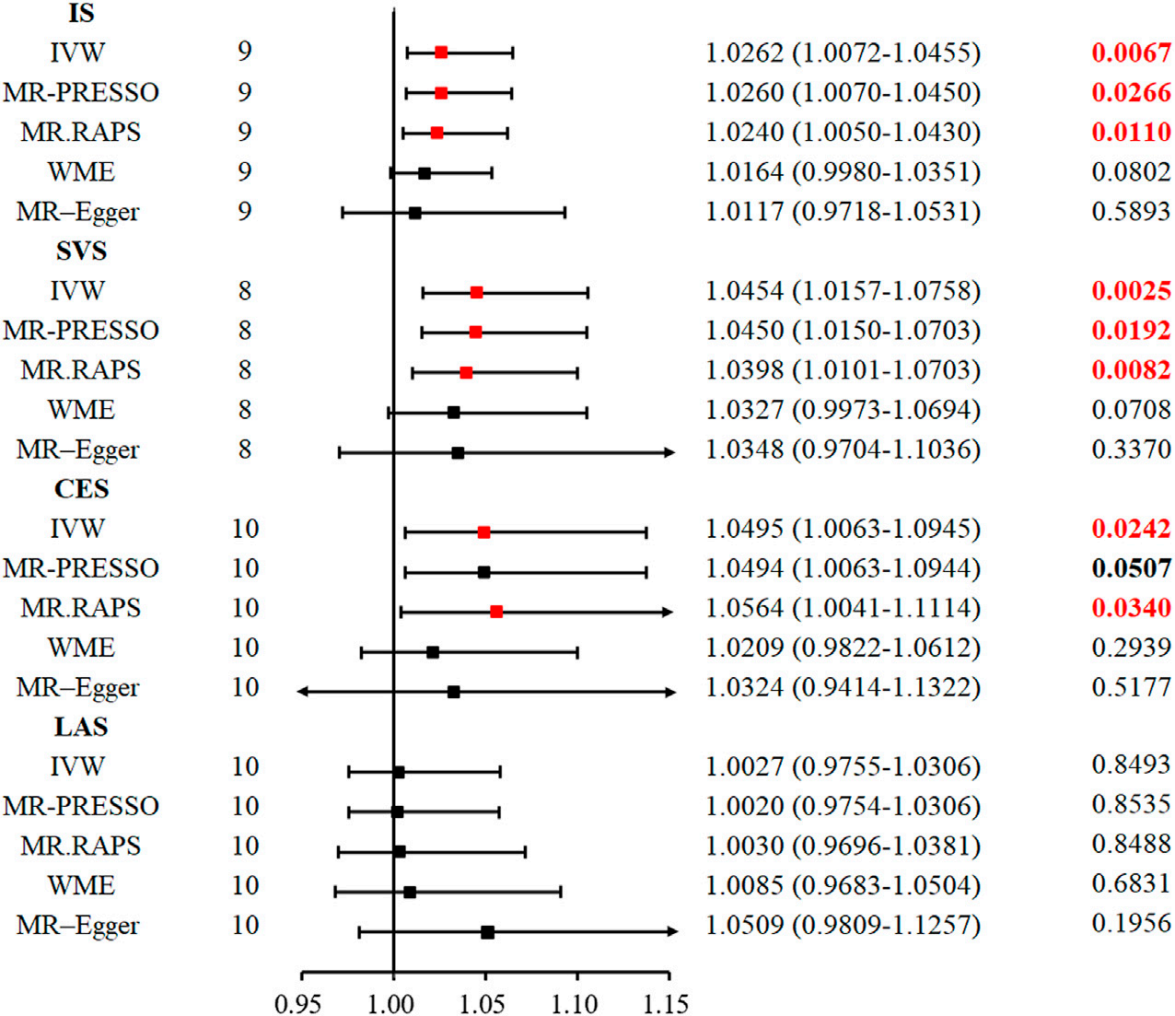
Five methods to assess the causal role of TIMP-3 in the risk of IS and its subtype. CI = confidence interval, IVW = inverse-variance-weighted, MR = Mendelian randomization, OR = odds ratio, SNP = single nucleotide polymorphism, IS = ischemic stroke, LAS = large vessel ischemic stroke, CES = cardioembolic ischemic stroke, SVS = small vessel ischemic stroke, MR. RAPS = MR-Robust Adjusted Profile Score, MR-PRESSO = MR pleiotropy residual sum and outlier.

**FIGURE 3:**
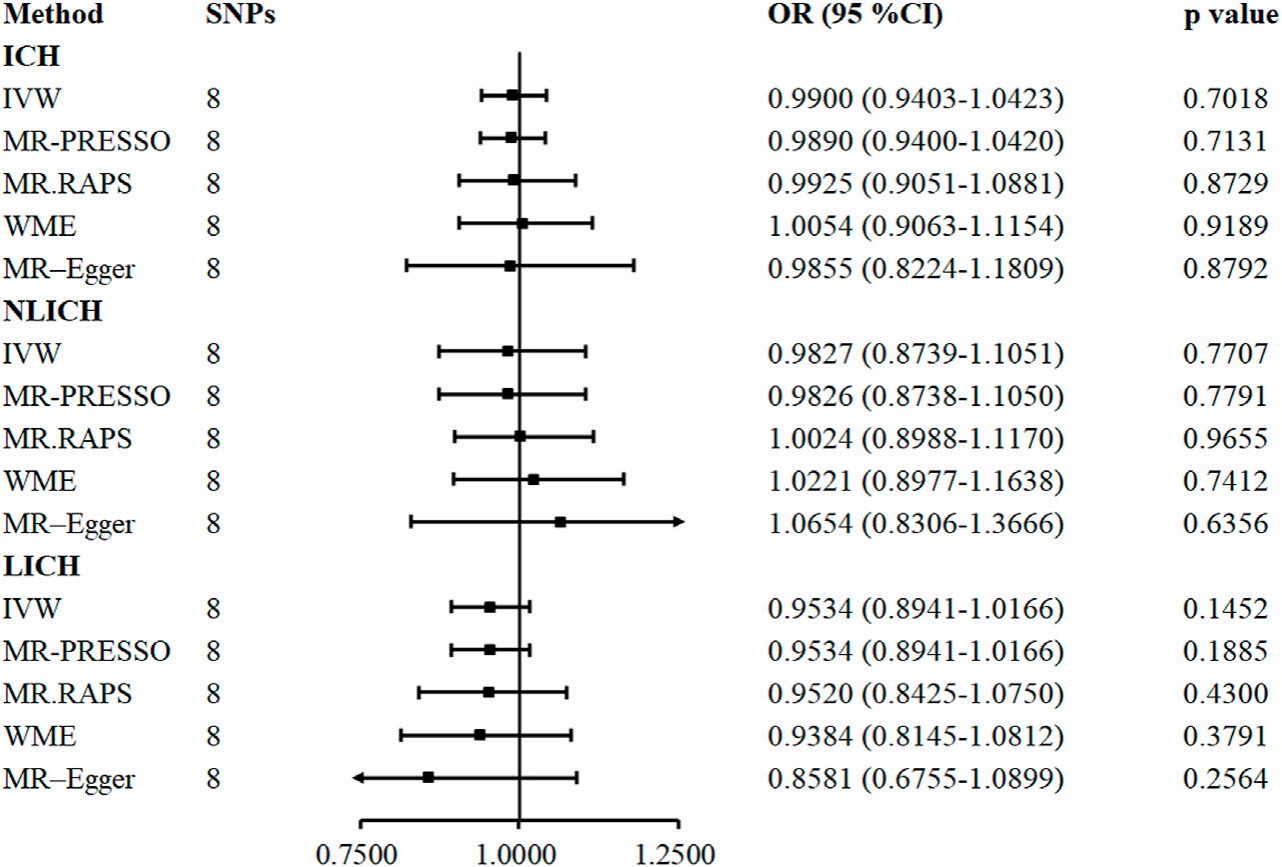
Five methods to assess the causal role of TIMP-3 in the risk of ICH and its subtype. CI = confidence interval, IVW = inverse-variance-weighted, OR = odds ratio, SNP = single nucleotide polymorphism, ICH = intracerebral hemorrhage, NLICH = non-lobar intracerebral hemorrhage, LICH = lobar intracerebral hemorrhage. MR. RAPS = MR-Robust Adjusted Profile Score, MR-PRESSO = MR pleiotropy residual sum and outlier.

Genetically proxied serum TIMP-3 level was positively associated with IS, especially SVS. The odds ratio (OR) of the genetically evaluated causal role of TIMP-3 in the risk of IS was 1.0262 (95% CI: 1.0072–1.0455, *p* = 0.0067) in the random-effect IVW model (Figure 2; Figure 3). There was also robust evidence of a positive correlation in MR. RAPS (OR = 1.0240, 95% CI: 1.0050–1.0430, *p* = 0.011) and MR-PRESSO (OR = 1.0260, 95% CI: 1.0070–1.0450, *p* = 0.0266). However, lower precision was found in WME (OR = 1.0164, 95% CI: 0.9980–1.0351, *p* = 0.0801) and MR-Egger regression (OR = 1.0117, 95% CI: 0.9718–1.0531, *p* = 0.589).

Likewise, in the SVS subtype, the random-effect IVW model suggested that increased serum TIMP-3 increases the risk of SVS (OR = 1.0454, 95% CI: 1.0157–1.0758, *p* = 0.0025). The MR. RAPS (OR = 1.0398, 95% CI: 1.0101–1.0703, *p* = 0.0082) and MR-PRESSO (OR = 1.0450, 95% CI: 1.0150–1.0703, *p* = 0.0192) approaches also supported the causal relationship between serum TIMP-3 and SVS. We found weak evidence for the relationship between TIMP-3 and the risk of CES (OR = 1.0495, 95% CI: 1.0063–1.0945, *p* = 0.024; Figure 2 and Supplementary Figure S1). However, after Bonferroni correction for multiple comparisons, the relationship was not significant. The causal role of serum TIMP-3 in LAS (OR = 1.0027, 95% CI: 0.9755–1.0306, *p* = 0.849) could not be confirmed by MR analysis.

### The Genetic Loci With Strongest Magnitude of Association With Ischaemic Stroke, Small Vessel Stroke, and Cardioembolic Stroke

In addition to finding a causal relationship between TIMP-3 and IS and its subtypes, we also identified genetic loci closely associated with TIMP-3, which may help with the prevention of IS in the future. Among them, two genetic loci, rs135150 and rs3788495 were positively related to IS. Moreover, rs135150 was also associated with SVS. Four genetic loci, rs135150, rs34843069, rs3788495, and rs241890 were positively associated with CES (Supplementary Figure S2).

### *TIMP-3* Does not Play a Causal Role in Intracerebral Haemorrhage Subtypes

In contrast to IS, the causal correlation between serum TIMP-3 and ICH was not found by IVW (OR = 0.9900, 95% CI: 0.9403–1.0423, *p* = 0.701), MR. RAPS (OR = 0.9925, 95% CI: 0.9051–1.0881, *p* = 0.8729), MR-PRESSO (OR = 0.9890, 95% CI: 0.9400–1.0420, *p* = 0.7131), WME (OR = 1.0054, 95% CI: 0.9063–1.1154, *p* = 0.918), or MR-Egger regression (OR = 0.9855, 95% CI: 0.8224–1.1809, *p* = 0.8791). The genetically predicted causal role of TIMP-3 in ICH subtypes was similar to that in ICH, and no causal relationship was observed in any one of the five models (Figure 3).

### Heterogeneity Test and Pleiotropy Test

As shown in Table 1, heterogeneity was not found in ICH (*p* = 0.936) or in either of its subtypes, LICH (*p* = 0.954) and NLICH (*p* = 0.263). In ischaemic stroke and its subtypes, we could not identify heterogeneity in IS (*p* = 0.069), LAS (*p* = 0.709), or SVS (*p* = 0.385), but heterogeneity was revealed in the CES subtype (*p* = 0.022). Therefore, the random effects model, IVW, was implemented in this study to analyse causality.

**TABLE 1:** Heterogeneity tests in the causality of TIMP-3 and IS, ICH. Q = Cochran’s Q statistic; df = degrees of freedom. IVW = inverse-variance-weighted, WME = Weighted median estimation, IS = ischemic stroke, LAS = large vessel ischemic stroke, CES = cardioembolic ischemic stroke, SVS = small vessel ischemic stroke, ICH = intracerebral hemorrhage, NLICH = non-lobar intracerebral hemorrhage, LICH = lobar intracerebral hemorrhage.

Outcome	Heterogeneity Test			
	Method	Q	Q_df	*p*-Value
IS				
	IVW	14.5	8	0.069
	WME	13.3	7	0.065
SVS				
	IVW	7.43	7	0.385
	WME	7.28	6	0.295
CES				
	IVW	19.4	9	0.022
	WME	19.1	8	0.015
LAS				
	IVW	6.31	9	0.709
	WME	3.99	8	0.857
ICH				
	IVW	2.37	7	0.936
	WME	2.37	6	0.882
NLICH				
	IVW	8.86	7	0.263
	WME	8.14	6	0.228
LICH				
	IVW	2.10	7	0.954
	WME	1.12	6	0.980

The pleiotropy of causality in TIMP-3 with IS (intercept = 0.0077, *p* = 0.454) and ICH (intercept = 0.0026, *p* = 0.956) were not detected in the MR-Egger regression (Table 2). Moreover, in the MR-PRESSO global test, the pleiotropy of the causal relationship between TIMP-3 and IS (*p* = 0.134) and ICH (*p* = 0.945) was also not evidenced.

**TABLE 2:** Pleiotropy Test in the causality of TIMP-3 and IS, ICH., IS = ischemic stroke, LAS = large vessel ischemic stroke, CES = cardioembolic ischemic stroke, SVS = small vessel ischemic stroke, ICH = intracerebral hemorrhage, NLICH = non-lobar intracerebral hemorrhage, LICH = lobar intracerebral hemorrhage.

Outcome	Pleiotropy Test				
	MR-Egger			MR-PRESSO	
	Egger Intercept	SE	*p*-Value	Global test	*p*-Value
IS	0.0077	0.0097	0.454	21.75	0.134
SVS	0.0055	0.0158	0.737	10.25	0.428
CES	0.0089	0.0224	0.702	27.70	0.082
LAS	-0.0258	0.0169	0.167	7.69	0.721
ICH	0.0026	0.0459	0.956	2.99	0.945
NLICH	-0.0461	0.0632	0.493	12.02	0.301
LICH	0.0598	0.0604	0.360	2.75	0.963

## Discussion

We performed a comprehensive MR analysis to investigate the causal role of serum TIMP-3 levels in the occurrence of IS, ICH, and their various subtypes. We found that TIMP-3 played a causal role in the incidence of IS, especially in SVS; however, no significant evidence was found to support a causal relationship between TIMP-3 and any ICH subtype. Moreover, there was no significant heterogeneity or pleiotropy, which confirmed the validity of our conclusions.

To the best of our knowledge, there are no published observational or MR studies on the relationship between TIMP-3 and IS and ICH in the literature. However, it is widely known that TIMP-3, a member of the regulatory matrix metalloenzyme family, plays a crucial role in regulating the restructuring of vascular ECM. To provide evidence of a causal relationship between TIMP-3 and IS, ICH, and their subtypes; control potential confounding factors; and reduce the occurrence of reverse causation, we performed a two-sample MR analysis. In the present study, we demonstrated that TIMP-3 played a causal role in the development of IS and its subtype, SVS and CES. This outcome may be a vital entry point for investigating the impact of MMPs on IS, especially SVS.

The ECM structure is regulated by the dynamic balance of TIMPs and MMPs, which restructure the ECM by regulating transcription mediated by cytokines and growth factors or by interfering with RNA at the transcriptional level (Brew and Nagase, 2010; Carreca et al., 2020). Interestingly, the dynamic balance between secreted MMPs and TIMPs is closely regulated by the molecules’ affinities for the low-density lipoprotein receptor-related protein-1 (LRP-1) (Hahn-Dantona et al., 2001; Thevenard et al., 2014; Gilardoni et al., 2017; Carreca et al., 2020). Therefore, the affinity of LRP-1 for MMPs and TIMPs is a substantial regulator of the dynamic structure of the ECM (Carreca et al., 2020). Furthermore, previous studies have confirmed that TIMP-3 has the strongest affinity for LRP-1 among all TIMPs (Carreca et al., 2020). TIMP-3 also promotes LRP-1-mediated-endocytosis by enhancing the binding of target metalloproteinases to LRP-1, which may aggravate cerebral vascular injury. Altogether, the LRP-1–TIMPs/MMPs pathway may contribute to the mechanisms of IS and SVS injury triggered by TIMP-3.

Incidence of SVS is closely related to hypertension. A previous study found that among all *TIMP* knockout mice, only *TIMP-3* knockout mice inhibited angiotensin II-induced blood pressure elevation. This supports a significant role for TIMP-3 in regulating hypertension (Basu et al., 2013). TIMP-3 can inhibit MMP-2, MMP-3, and MMP-9 which regulate ECM structure and vascular remodelling and consequently affect hypertension-induced stroke (Jin et al., 2017; Li et al., 2019b). Therefore, the increased risk of IS, especially SVS, due to TIMP-3 may be highly correlated to the regulation of MMPs by TIMP-3.

In addition, previous research suggests that TIMP-3 is a potent angiogenesis inhibitor that suppresses vascular endothelial growth factor (VEGF)-mediated angiogenesis which affects vascular repair and leads to cumulative blood vessel damage. This effect is more pronounced in small blood vessels (Qi et al., 2003; Janssen et al., 2008). Moreover, it has been suggested that TIMP-3 also plays an important role in the myogenic autoregulation of arterioles. *TIMP-3* overexpression blocks the ADAM17 (a disintegrin and metalloprotease 17)/HB-EGF (heparin-binding EGF-like growth factor)/(ErbB1/ErbB4) pathway, which weakens the myogenic regulation of small vessels and makes it difficult for vessels to recover quickly (Chalaris et al., 2010; Xu et al., 2012; Capone et al., 2016). The combination of these effects can exacerbate the occurrence of small vascular stroke. Previous studies have shown that LAS is more susceptible to atherosclerosis, and CES is most closely related to cardiogenic emboli and atrial fibrillation. The effect of TIMP-3 on the atherosclerosis, cardiogenic emboli and atrial fibrillation was weak. This may be one reason that TIMP-3 is more related to SVS than other IS subtypes.

This study mainly focused on European populations with the advantage of ruling out the impact of population differences on the study findings. It is based on published large sample genetics data and takes multiple approaches to explore causality without significant sensitivity and heterogeneity concerns which produced robust and reliable results.

Some limitations of the study are open to discussion here. First, it is difficult to exclude the effects of directional pleiotropy, which makes exploring causality prone to bias. However, in our study neither MR-Egger regression nor MR-PRESSO Global test showed statistically significant directional pleiotropy, ruling out the influence of directional pleiotropy. Moreover, because of the small number of instrumental variables identified, we adopted a more relaxed threshold (*p* < 1 × 10^–5^) to explore the causal relationship between TIMP-3 and IS/ICH; however, the reliability of this relaxation has been confirmed by other studies (Sun et al., 2020). Thirdly, the Mendelian randomisation approach offers a relatively weak genetic interpretation, which resulted in the low OR value for causality; however, experimentally confirmed values have been obtained in previous studies (Georgakis et al., 2019). Finally, our approach to screening for instrumental variables used the screening criteria of GWAS studies without incorporating cis expression quantitative trait loci (pQTL). We performed rigorous testing for pleiotropy of exposure and outcome using MR-Egger regression and MR-PRESSO methods, but no pleiotropy was found, which suggests that our results are robust. In future MR studies, combining cis pQTL and traditional methods to screening instrumental variables may find more precise biomarkers.

## Conclusion

In summary, our MR study provides positive evidence for the causal role of TIMP-3 in the risk of developing IS, especially SVS. We found two SNP loci, rs135150 and rs3788495, which may be important biomarkers for predicting the occurrence of IS. The underlying mechanism in the causal relationship between TIMP-3 and the risk of IS and SVS warrants further research and may have vital clinical implications in the prevention of IS.

## Data Availability

The original contributions presented in the study are included in the article/Supplementary Material, further inquiries can be directed to the corresponding author.
