# Supplementary material for: Evaluating the Causal Effects of TIMP-3 on Ischaemic Stroke and Intracerebral Haemorrhage: A Mendelian Randomization Study
Source: Front Genet. 2022 Apr 4;13:838809. doi: 10.3389/fgene.2022.838809 (PMC9015162; doi:10.3389/fgene.2022.838809)
Supplement: Supplementary file 2 [file Table1.DOCX]

**Table 1: Heterogeneity tests in the causality of TIMP-3 and IS, ICH.**

Q=Cochran’s Q statistic; df=degrees of freedom. IVW = inverse-variance-weighted, WME=Weighted median estimation, IS= ischemic stroke, LAS= large vessel ischemic stroke, CES= cardioembolic ischemic stroke, SVS=small vessel ischemic stroke, ICH=intracerebral hemorrhage, NLICH=non-lobar intracerebral hemorrhage, LICH= lobar intracerebral hemorrhage.

| **Heterogeneity Test** | | | | |
| --- | --- | --- | --- | --- |
| **Outcome** | **Method** | **Q** | **Q_df** | **p-Value** |
| **IS** |  |  |  |  |
|  | IVW | 14.5 | 8 | 0.069 |
|  | WME | 13.3 | 7 | 0.065 |
| **SVS** |  |  |  |  |
|  | IVW | 7.43 | 7 | 0.385 |
|  | WME | 7.28 | 6 | 0.295 |
| **CES** |  |  |  |  |
|  | IVW | 19.4 | 9 | 0.022 |
|  | WME | 19.1 | 8 | 0.015 |
| **LAS** |  |  |  |  |
|  | IVW | 6.31 | 9 | 0.709 |
|  | WME | 3.99 | 8 | 0.857 |
| **ICH** |  |  |  |  |
|  | IVW | 2.37 | 7 | 0.936 |
|  | WME | 2.37 | 6 | 0.882 |
| **NLICH** |  |  |  |  |
|  | IVW | 8.86 | 7 | 0.263 |
|  | WME | 8.14 | 6 | 0.228 |
| **LICH** |  |  |  |  |
|  | IVW | 2.10 | 7 | 0.954 |
|  | WME | 1.12 | 6 | 0.980 |
