# Supplementary material for: Evaluating the Causal Effects of TIMP-3 on Ischaemic Stroke and Intracerebral Haemorrhage: A Mendelian Randomization Study
Source: Front Genet. 2022 Apr 4;13:838809. doi: 10.3389/fgene.2022.838809 (PMC9015162; doi:10.3389/fgene.2022.838809)
Supplement: Supplementary file 3 [file Table2.DOCX]

**Table 2: Pleiotropy Test in the causality of TIMP-3 and IS, ICH.**

, IS= ischemic stroke, LAS= large vessel ischemic stroke, CES= cardioembolic ischemic stroke, SVS=small vessel ischemic stroke, ICH=intracerebral hemorrhage, NLICH=non-lobar intracerebral hemorrhage, LICH= lobar intracerebral hemorrhage.

| **Pleiotropy Test** | | | | | | | |
| --- | --- | --- | --- | --- | --- | --- | --- |
| Outcome |  | **MR-Egger** | | |  | **MR-PRESSO** | |
|  |  | Egger Intercept | SE | p-Value |  | Global test | p-Value |
| **IS** |  | 0.0077 | 0.0097 | 0.454 |  | 21.75 | 0.134 |
| SVS |  | 0.0055 | 0.0158 | 0.737 |  | 10.25 | 0.428 |
| CES |  | 0.0089 | 0.0224 | 0.702 |  | 27.70 | 0.082 |
| LAS |  | -0.0258 | 0.0169 | 0.167 |  | 7.69 | 0.721 |
| **ICH** |  | 0.0026 | 0.0459 | 0.956 |  | 2.99 | 0.945 |
| NLICH |  | -0.0461 | 0.0632 | 0.493 |  | 12.02 | 0.301 |
| LICH |  | 0.0598 | 0.0604 | 0.360 |  | 2.75 | 0.963 |
